# Nephrolithiasis risk factors for obese patients on 24‐hour urine collection metabolic evaluation

**DOI:** 10.1002/bco2.70103

**Published:** 2025-10-30

**Authors:** Mark I. Sultan, Satoshi Yamazaki, Shady A. Ibrahim, Hadeel Haddad, Antoinette Abdelmalek, Sohrab N. Ali, Mac Kinnly T. Knoerzer, Muhammed A. M. Hammad, Ramy F. Youssef

**Affiliations:** ^1^ Department of Urology University of California Irvine CA USA

**Keywords:** 24 hour urine collection, nephrolithiasis risk factors, obesity and nephrolithiasis, stone metabolic evaluation, urinary calcium stones

## Abstract

**Objectives:**

Twenty‐four‐hour urine collections are obtained as part of the metabolic workup for nephrolithiasis to identify modifiable abnormalities for stone prevention. We sought to discern trends in the prevalence of abnormalities based on body mass index (BMI) (kg/m^2^).

**Material and Methods:**

All unique Litholink™ 24‐Hour Urine outcomes for nephrolithiasis prior to medical or dietary therapy obtained at our institution between 2004 and 2020 were retrospectively reviewed. Patients with anthropometric data were classified according to body mass index (BMI) as underweight (<18.5), normal (18.5–25), overweight^25‐30^ and obese (>30). Litholink™ 24‐Hour Urine gender specific reference ranges were used to define abnormalities.

**Results:**

A total of 1372 patients were included. The mean age was 56 ± 15.3 (53.5% male, 46.5% female). Cumulatively, 30.7% (421/1372) were obese, 32.9% (452/1372) overweight, 33.4% (458/1372) normal and 3.0% (41/1372) underweight. Overall, elevated urine sodium was the most common metabolic abnormality (52.8%). In obese stone formers, hypercalciuria (p = 0.027), hyperoxaluria (p < 0.001), elevated urine sodium (p < 0.001), hyperuricosuria (p < 0.001) low urine pH (p < 0.001) and high uric acid supersaturation (p < 0.001) were more likely compared to normal BMI stone formers. Underweight patients demonstrated greater likelihood of oliguria (p = 0.001), without adjustment for weight, and hypocitraturia (p = 0.001) compared to normal BMI stone formers.

**Conclusions:**

Body weight differences are associated with different risk profiles on 24‐hour urine collection. Obese, and to a letter degree, underweight patients are more likely to harbour metabolic derangements on a 24‐hour urine analysis compared to patients with normal BMI, thus underscoring the importance of directed medical therapy for stone formers.

## 1 INTRODUCTION

Nephrolithiasis, or kidney stone disease, persists as a widespread global issue, with a noticeable increase in disease incidence over recent decades. According to data extrapolated from the National Health and Nutrition Examination Survey (NHANES), the prevalence of stone disease in the United States has risen from 3.2% in 1980 to 11.0% between 2015 and 2018.^1^, ^2^ This rise in prevalence has been linked to an increase in obesity and changes in dietary habits, particularly as there has been a parallel growth in the obesity rate while previous studies have demonstrated a significant association between obesity and nephrolithiasis.^3^, ^4^, ^5^

Kidney stones are complex crystalline structures originating from a diverse composition of mineral deposits within the renal calyces and pelvis.^6^ The most common types of kidney stones are calcium‐based, formed when calcium binds to oxalate due to factors such as low urine volume, hypercalciuria, hypocitraturia or hyperoxaluria.^7^ Studies have shown that obese patients are more likely to have increased urinary calcium, oxalate and uric acid excretion, thereby increasing their risk for calcium‐based kidney stones.^8^ Meanwhile, uric acid stones, a byproduct of uric acid crystal accumulation in a low pH environment, are also linked to conditions such as obesity, diabetes and gout.^9^

In recent years, the American Urologic Association (AUA) treatment paradigm advocates for metabolic testing to delineate modifiable risk factors for patients with recurrent stone disease. The prevailing metabolic evaluation occurs as one or two 24‐hour urine collections to assess total urine volume, urine pH, calcium, oxalate, uric acid, citrate, sodium, potassium and creatinine.^10^ However, the prevalence of metabolic abnormalities appears impacted by a multitude of demographic factors, including BMI, further obscuring the aetiology of modifiable risk factors for patients with nephrolithiasis. We aim to further delineate potential differences in nephrolithiasis risk factors as BMI increases with a retrospective review of all initial 24‐hour urine collections performed at our tertiary academic centre.

## 2 MATERIALS AND METHODS

### 2.1 Patient population and classification

The Institutional Review Board (IRB) of the University of California, Irvine approved this study (#3425). All Litholink™ (Itasca, IL, USA) 24‐Hour Urine collections for a nephrolithiasis metabolic evaluation obtained at our institution between 2004 and 2020 were retrospectively reviewed. Only initial 24‐hour urine collections prior to medical or lifestyle/dietary intervention for each patient were included in the analysis. Cysteine stone formers or patients with cystinuria were excluded from the cohort to comprise either calcium or uric acid stone formers. Stone risk factors obtained from the Litholink™ 24‐Hour Urine report includes urine volume, calcium, oxalate, citrate, sodium, uric acid and urine pH in addition to the supersaturation of calcium oxalate, calcium phosphate and uric acid. Litholink™ 24‐Hour Urine gender specific reference ranges were used to define abnormalities and are in Table S1. Low urine volume was defined as a 24‐hour urine volume below 1.5 L and a pH below 5.5 was considered as very acidic urine. For the purposes of analysis, the patient cohort included only those with available anthropometric data and were stratified by BMI (kg/m^2^) as underweight (< 18.5), normal (18.5–24.9), overweight (25–29.9) or obese (≥ 30). A total of 1372 patients met inclusion criteria for analysis.

### 2.2 Statistical analysis

Descriptive statistics for continuous variables were reported as a median value. After assessing the normality by a Kolmogorov–Smirnov test, a Kruskal–Wallis test was used to compare the distribution of continuous variables between different BMI groups. Descriptive statistics for categorical variables were expressed as a number and proportion of patients. A Chi‐Square test was used determine the significance of variability for dichotomous or multi‐level categorical grouping. Odds ratios between BMI groups were calculated using a Mantel–Haenszel common odds ratio estimate and reported with a 95% confidence interval. Further, a multivariate binary logistic regression model using age and gender covariates with BMI group was performed on urinary abnormalities to identify the significance of demographic factors attributable risk on a particular urine analyte. Age was modelled as a continuous independent predictor, gender as a categorical independent predictor, and the BMI group as a categorical independent predictor with the urinary abnormality as a binary outcome. All analyses were performed using SPSS software version 29.0 (IBM Corp. Armonk, NY) and p < 0.05 was considered statistically significant.

## 3 RESULTS

### 3.1 Patients demographics

A total of 1372 patients met inclusion criteria for analysis to include 638 (46.5%) females and 734 (53.5%) males with an average age of 56 ± 15. BMI was not uniformly distributed in this cohort with 41 (3.0%) patients considered underweight, 458 (33.4%) patients considered normal weight, 452 (32.9%) patients considered overweight and 421 (30.7%) patients considered obese. Obese individuals were subclassified into class I (BMI 30–34.9), class II (BMI 35.0–39.9) or class III (BMI ≥ 40) which were comprised of 240 (57.0%), 92 (21.9%) and 89 (21.1%) patients, respectively, yielding an uneven distribution favouring patients of class I obesity (p = 0.000003) (Table S2). A larger proportion of male patients were considered either overweight or obese (67.3%) compared to the proportion of either overweight or obese female patients (59.4%) (p = 0.002). Patient demographics are included in Table 1.

**TABLE 1:** Cohort demographics of stone forming patients evaluated via Litholink™ 24 hour urine testing from 2004 to 2020 at a tertiary care center in Southern California.

Gender		BMI group				
		Underweight	Normal	Overweight	Obese	Total
Female	N	28	231	167	212	638
	%	4.4%	36.2%	26.2%	33.2%	100.0%
	Mean Age (SD)	54 (18)	52 (16)	57 (14)	55 (15)	55 (15)
Male	N	13	227	285	209	734
	%	1.8%	30.9%	38.8%	28.5%	100.0%
	Mean Age (SD)	46 (22)	57 (18)	59 (13)	57 (13)	57 (15)
Total	N	41	458	452	421	1372
	%	3.0%	33.4%	32.9%	30.7%	100%

Total count (N) and proportion (%) of gender distribution in addition to the mean and standard deviation (SD) age stratified by BMI group.

### 3.2 Differences in metabolic risk factors based on BMI classification

All analytes varied significantly with respect to BMI classification. The median analyte value and significance in variation among BMI classes are reported in Table 2. Table 3 details the prevalence of urinary metabolic abnormalities across BMI groups. Urinary calcium, oxalate, sodium, uric acid and urine pH measurements demonstrate consistent yet progressive escalation in risk as BMI increases (Table 2).

**TABLE 2:** Median analyte value and significance in variation among different BMI classes.

BMI group					
Urine Analyte	Underweight	Normal	Overweight	Obese	Sig.
Volume (L/Day)	**1.45 (** ±0.84)	2.12 **(** ±0.89)	2.02 **(** ±0.87)	1.93 **(** ±0.85)	**0.008**
Calcium (mg/Day)	144.90 **(** ±68.73)	172.16 **(** ±86.23)	182.20 **(** ±99.37)	**185.45 (** ±123.58)	**0.001**
Oxalate (mg/Day)	27.63 **(** ±15.97)	32.28 **(** ±18.03)	35.43 **(** ±14.14)	**37.81 (** ±18.53)	**P < 0.001**
Citrate (mg/Day)	**282.62 (** ±210.12)	446.05 **(** ±272.60)	523.05 **(** ±302.50)	518.74 **(** ±390.81)	**P < 0.001**
Sodium (mmol/Day)	91.39 **(** ±48.27)	135.15 **(** ±57.45)	160.50 **(** ±56.72)	**177.55 (** ±74.91)	**P < 0.001**
Uric Acid (g/Day)	0.356 **(** ±0.16)	0.547 **(** ±0.19)	0.596 **(** ±0.21)	**0.662 (** ±0.27)	**P < 0.001**
pH	6.36 **(** ±0.73)	6.30 **(** ±0.53)	6.09 **(** ±0.50)	**5.92 (** ±0.52)	**P < 0.001**
CaOx Supersaturation	5.72 **(** ±3.58)	5.16 **(** ±3.43)	5.52 **(** ±2.98)	**5.94 (** ±2.76)	**0.048**
CaP Supersaturation	**0.940 (** ±0.79)	0.843 **(** ±0.75)	0.835 **(** ±0.84)	0.639 **(** ±0.78)	**0.016**
UA Supersaturation	0.270 **(** ±0.70)	0.328 **(** ±0.54)	0.522 **(** ±0.69)	**0.751 (** ±0.76)	**P < 0.001**

Distribution of continuous variables between different BMI groups assessed by the Kruskal‐Wallis test. The median value is reported. CaOx: Calcium Oxalate, CaP: Calcium Phosphate, UA: Uric Acid.

**TABLE 3:** Prevalence of urinary metabolic abnormality across BMI classes.

BMI group					
Urine abnormality	Underweight	Normal	Overweight	Obese	All
Low Volume	**51.2%**	26.4%	30.3%	28.3%	29.0%
Hypercalciuria	14.6%	33.6%	34.7%	**40.9%**	35.6%
Hyperoxaluria	29.3%	29.1%	37.9%	**43.2%**	36.3%
Hypocitraturia	**85.4%**	57.6%	43.1%	48.7%	50.9%
Elevated Sodium	9.8%	41.3%	57.3%	**64.6%**	52.8%
Hyperuricosuria	4.9%	14.3%	23.2%	**34.1%**	23.0%
Low pH	14.6%	8.3%	13.5%	**25.9%**	15.6%
High Risk CaOx SS	14.6%	12.8%	**15.6%**	12.0%	13.5%
High Risk CaP SS	12.2%	14.1%	**15.2%**	12.9%	14.0%
High Risk UA SS	19.5%	19.2%	30.6%	**43.2%**	30.3%

CaOx SS: Calcium Oxalate Supersaturation, CaP SS: Calcium Phosphate Supersaturation, UA SS: Uric Acid Supersaturation.

Obese patients were more likely to harbour a multitude of metabolic risk factors compared to normal weight patients. For example, obese patients demonstrated a 40.9% prevalence for hypercalciuria compared to 33.6% of normal weight patients (OR = 1.36 [1.04–1.79], p = 0.027). In addition, 43.2% of obese patients developed hyperoxaluria compared to 29.1% of normal patients (OR = 1.85 [1.40–2.45], p < 0.001). Similarly, 64.6% of obese patients presented with elevated urine sodium compared to 41.3% of normal weight patients (OR = 2.60 [1.98–3.41], P < 0.001). Greater prevalence of hyperuricosuria was observed among obese patients at 34.1% compared to 14.3% of normal patients (OR = 3.09 [2.22–4.31], P < 0.001). Lastly, obese patients were more likely to yield a urine pH below 5.5 with a corresponding prevalence of 25.9% compared to 8.3% of normal weight patients (OR = 3.86 [2.60–5.75], P < 0.001). Concordantly, obese patients were more likely to demonstrate a high‐risk uric acid supersaturation demonstrating a prevalence of 43.2% compared to 19.2% of normal weight patients (OR = 3.20 [2.37–4.34], P < 0.001).

On group analysis, underweight patients were significantly more likely to demonstrate a low urine volume with a prevalence of 51.2% compared to the prevalence of low urine volume in normal patients of 26.4% (OR = 2.94 [1.53–5.58], P = 0.001). Interestingly, when urine volume was normalized by Kg of body weight, obese patients exhibited a 17.1% prevalence for oliguria, defined as less than 0.5 ml/kg/h, compared to 4.1% of normal weight patients (OR = 4.767 [2.82–8.06], P < 0.001). Meanwhile only 2.4% of underweight patients demonstrated oliguria. However, underweight patients were more likely to present with hypocitraturia, noting a prevalence of 85.4% compared to 57.6% of normal weight patients (OR = 4.29 [1.77–10.39], P = 0.001).

### 3.3 Multivariable model for demographic predictors on metabolic outcomes for nephrolithiasis

A multivariate logistic regression accounting for patient age, sex and BMI group was performed to further delineate the robustness of demographic predictors for a urinary analyte abnormality. The significance of each demographic predictor coefficient in the multivariate model is included in Table S3. Regarding low urine volume, only gender remained a significant predictor after accounting for other demographic classifications (P < 0.001). Otherwise, BMI differences remained a significant predictor for hypercalciuria, hyperoxaluria, hypocitraturia, elevated urine sodium, hyperuricosuria and low urine pH, thereby demonstrating an association after accounting for age and gender differences.

## 4 DISCUSSION

Obesity, as measured by BMI, is a recognized risk factor for stone disease, emphasizing the importance of weight management in stone prevention.^11^ The results of this analysis are poised to reinforce existing nuances in assessable risk factors for nephrolithiasis based on a 24‐hour urine collection as they relate to patients of different body habitus within a real clinical practice setting. In our analysis, increasing BMI demonstrated a stepwise increase in the risk for hypercalciuria, hyperoxaluria, an elevated urine sodium, hyperuricosuria and low urine pH. Our results mirror previously published studies including a retrospective review of 1698 stone forming patients demonstrating a higher urinary excretion of calcium, oxalate and urate in overweight and obese patients compared to normal patients.^12^ Previous studies have also shown that urine pH demonstrated an inverse correlation with body weight, fat mass and percentage of body fat among patients with kidney stones.^13^ Notably, these associations with BMI class were observed despite a significant majority of obese patients presenting with milder class I obesity as opposed to class II and class III obesity.

Although there are different crystalline stone compositions, nearly 80% of all removed or excreted renal calculi are calcium‐based.^14^ Various pathophysiologic mechanisms for hypercalciuria exist, including increased intestinal calcium absorption, enhanced calcium mobilization from the bones and reduced renal tubular calcium reabsorption. Particularly for obese patients, previous evidence has demonstrated that an increase in serum insulin or insulin sensitivity may contribute to an exaggerated calciuric response, and thus propagate the pathogenesis of calcium stone disease.^15^ However, more recent evidence comparing patients with idiopathic hypercalciuria to non‐stone formers of similar BMI and insulin sensitivity demonstrated a nonsignificant impact of serum insulin on postprandial hypercalciuria regardless of body weight classification, therefore attributing dietary factors such as a higher salt and animal protein intake as predictors of urinary calcium excretion.^15^, ^16^ Meanwhile the causes of hyperoxaluria include increased consumption of oxalate rich foods, increased intestinal oxalate absorption and increased hepatic oxalate synthesis due to genetic errors in metabolism.^17^ Epidemiologic data has shown calcium oxalate stones to be the most common stone composition, and given that there has been a gradual increase in the prevalence of hyperoxaluria among stone formers from 24.8% between 1980 and 1990 to over 45% between 2001 and 2013, it is likely that hyperoxaluria constitutes a portion of the etiological puzzle for stone prevalence, particularly in the obese population as previous work has demonstrated an increased incidence of hyperoxaluria among patients in this demographic.^18^, ^19^ However, in the setting of obesity, other comorbidities may confound the relationship to stone disease. In particular, the increased incidence of type 2 diabetes and gout in overweight or obese patients both contribute to stone formation.^20^ The normal physiologic effects of insulin include stimulatation of ammoniagenesis and subsequent enchanced Na+/H + exchange in the proximal tubule of the nephron. Therefore, insulin resistance promotes uric acid nephrolithiasis by inducing excess urine acidification.^21^

The diet of obese patients may differ from that of non‐obese patients, with greater sodium and animal protein intake while consuming decreased citrus fruits, vegetables and fibre.^22^ Calcium handling is dependent on sodium in the renal proximal tubule. Therefore, high dietary salt intake entails an elevated sodium load in the kidney which limits the efficacy of sodium and water reabsorption and, consequently, the reabsorption of calcium thereby leading to elevated urinary calcium excretion.^23^ In addition, excessive salt intake is associated with a lower upper limit of calcium oxalate metastability in urine, demonstrating that a high salt diet imparts a lithogenic effect beyond calciuric influence alone.^24^ As such, a higher salt intake has been associated with hypercalciuria and hypocitraturia, which are strong risk factors for calcium stone formation.^23^, ^25^ In conjunction, rising urinary acid excretion, a result of increased animal protein intake, is also accompanied by increased urinary calcium due to reduced tubular reabsorption.^26^ These dietary differences contribute clinically to the risk of stone disease as a diet low in animal protein, sodium, and oxalate with normal calcium intake in hypercalciuric men has previously demonstrated a reduction of nearly 50% in the recurrence of stone formation over 5 years.^27^ In addition, dyslipidemia has been linked to kidney stone formation accompanied by elevated risk in individuals who exhibit hypertriglyceridemia and low HDL‐cholesterol.^28^ Therefore, it is important to manage weight and dietary habits in kidney stone prevention, highlighting the necessity for routine urine monitoring, particularly as certain dietary habits have been demonstrated to limit the risk for stone disease.^7^ Additional factors have been demonstrated to influence urine metabolic abnormalities in nephrolithiasis patients. We previously queried the same database to examine urinary metabolite profiles stratified by age and gender which offers additional insight for results stratified by weight. Our analysis revealed that male patients presented with greater prevalence of high‐sodium and low‐pH urine, hyperuricosuria and uric acid supersaturation. Conversely, female patients exhibited greater prevalence of hypercalciuria, hypocitraturia and low urine volume thus demonstrating that gender and obesity independently demonstrate associations with specific urinary metabolic derangements.^29^ Therefore, the authors of this manuscript caution attributing the rise in prevalence of nephrolithiasis solely to a rise in obesity or an increased prevalence of metabolic risk factors on urine evaluation and recognize other contributors such as an increased availability of radiographic imaging and increased accessibility of healthcare to inflate the prevalence of stone disease in comparison to previous decades.^30^

While significant urinary metabolite abnormalities were demonstrated in obese nephrolithiasis patients, low urine output prior to weight adjustment and hypocitraturia were associated with underweight BMI. Although urinary output normalized upon weight adjustment, low output is strongly associated with insufficient calorie, protein, fibre fat and water intake thus suggesting a predisposition of patients of low BMI for oliguria.^31^ An association of oliguria with hypocitraturia has been demonstrated. Reduced nutrient intake may explain observed hypocitraturia in underweight patients as urinary citrate originates from dietary sources but may also be due to excess consumption of acid‐producing foods as metabolic acidosis promotes citrate resorption.^32^ Conversely, the association of oliguria after weight adjustment with obese individuals diverges from previous studies.^33^ Notably, this observation may be influence by the algorithm applied to adjust for body weight in which urine output was calculated using the actual body weight of the patient. In obese patients, calculation of urine output using actual body weight as opposed to ideal body weight is demonstrated to overestimate acute kidney injury.^34^ In turn, oliguria in our analysis may be falsely overestimated in obese patients.

As a retrospective analysis of patient data from a single institution, our study presents certain limitations. Patient selection was performed based on a historical diagnosis of kidney stones accompanied by a Litholink™ 24‐Hour Urine report which may reduce the generalizability of our findings to a broader population. The retrospective design further hindered control of covariates as genetic and functional risk factors for urolithiasis as well as other contributing factors such as dietary habits and family history of stone disease were not collected at the time of analysis. This dataset also includes idiopathic stone formers and patients who were not screened for the use of bisphosphonates, chronic nonsteroidal anti‐inflammatory drugs, glucocorticoids, anatomic urologic abnormalities, or other conditions that may raise the risk of stone disease. Furthermore, the heterogeneity of the practice patterns of the included clinicians contributed to a lack of standardization of the schedule of urine collection. The retrospective design further limited metrics for evaluation of the association of obesity and urinary metabolic abnormalities. Although BMI has been widely used as a metric to assess obesity, clinically defined as a condition manifested by the effects of excess adiposity on organs and tissues, its validity has attracted speculation. While its use in epidemiological studies as a metric for health risk due to obesity is considered reasonable, implementation on an individual level without the use of an additional anthropometric measurement is limited in its capacity to adjust account for muscle mass and fluid status.^35^ Therefore, using BMI as a metric of adiposity presents a greater likelihood erroneous classification of disease risk compared to approaches with greater accuracy in assessing adiposity. Lastly, we recognize inherent variations in 24‐hour urine collections revealed in recent literature which demonstrates that as many as 51% of 24‐hour urine collections at a tertiary care centre were performed inaccurately.^36^ Despite these limitations, this analysis serves to further validate existing differences in the prevalence of urinary risk factors attributable to body habitus in a clinical setting for a large contemporary cohort.

## 5 CONCLUSIONS

Metabolic factors for nephrolithiasis evaluated with 24‐hour urine collection metabolic evaluation demonstrate significant variability based on age, sex and BMI. Approximately 2/3 of stone patients are overweight or obese. Obese patients are more likely to harbour metabolic derangements on a 24‐hour urine analysis compared to normal BMI patients including hypercalciuria, an elevated urine sodium, hyperoxaluria, hypocitraturia, hyperuricosuria and a low urine pH. Furthermore, metabolic derangements were observed in underweight patients who demonstrated a greater likelihood of low urine volume without adjustment for weight and hypocitraturia compared to normal BMI stone formers. These variations underscore the importance of 24‐hour urine evaluation to tailor specific directed medical therapy in the prevention of nephrolithiasis. Limiting salt, oxalate rich food and moderating protein consumption may be effective preventative measures though further research is required to better understand the complex pathogenesis of nephrolithiasis and its relation to age, gender and BMI.

## CONFLICT OF INTEREST STATEMENT

Mark I. Sultan reports no conflicts of interest.

Satoshi Yamazaki reports no conflicts of interest.

Shady A. Ibrahim reports no conflicts of interest.

Hadeel Haddad reports no conflicts of interest.

Antoinette Abdelmalek reports no conflicts of interest.

Sohrab N. Ali reports no conflicts of interest.

MacKinnly T Knoerzer reports no conflicts of interest.

Muhammed A.M. Hammad reports no conflicts of interest.

Ramy F. Youssef reports no conflicts of interest.

## ETHICS STATEMENT

This study was approved by the Institutional Review Board of the University of California Irvine with approval number 3425.

## Supporting information


**Table S1.** Litholink™ 24 Hour Urine gender specific reference ranges.
**Supplementary Table 2.** Stratification of median analyte values by obesity class.
**Supplementary Table 3.** Relationship of age, gender and BMI with urinary analyte abnormalities on 24‐hour urine collection.
