# Supplementary material for: Nephrolithiasis risk factors for obese patients on 24‐hour urine collection metabolic evaluation
Source: BJUI Compass. 2025 Oct 30;6(11):e70103. doi: 10.1002/bco2.70103 (PMC12572948; doi:10.1002/bco2.70103)
Supplement: Supplementary file 1 — Table S1. Litholink™ 24 Hour Urine gender specific reference ranges. Supplementary Table 2. Stratification of median analyte values by obesity class. Supplementary Table 3. Relationship of age, gender and BMI with urinary analyte abnormalities on 24‐hour urine collection. [file BCO2-6-e70103-s001.docx]

Variations in the Prevalence of Nephrolithiasis Risk Factors for Obese Patients on 24-Hour Urine Collection Metabolic Evaluation

Mark I. Sultan, Satoshi Yamazaki, Shady A. Ibrahim, Sohrab N. Ali, & Ramy F. Youssef ^1^

Department of Urology, University of California, Irvine: Orange, CA 92868, USA

^1^[ryaacoub@hs.uci.edu](mailto:ryaacoub@hs.uci.edu)

**Supplementary Table 1.** Litholink™ 24 Hour Urine gender specific reference ranges.

| Reference Range | |
| --- | --- |
| Urine Analyte | Normal Range |
| Calcium | Male: < 250 mg/day  Female: < 200 mg/day |
| Oxalate | 20-40 mg/day |
| Citrate | Male: >450 mg/day  Female: > 550 mg/day |
| Sodium | 50-150 mmol/day |
| Uric Acid | Male: < 0.80 g/day  Female: < 0.75 g/day |
| SS Calcium Oxalate | 6-10 |
| SS Calcium Phosphate | 0.5-2 |
| SS Uric Acid | 0-1 |
| SS: Supersaturation | |

**Supplementary Table 2.** Stratification of median analyte values by obesity class

| BMI Group | | | |  |
| --- | --- | --- | --- | --- |
| Urine Analyte | Obesity I  (n=240) | Obesity II  (n=92) | Obesity III  (n=89) | Significance  **P=0.000003** |
| Volume (L/Day) | 2.13 (±0.80) | 2.09 (±0.87) | 2.31 (±0.96) |  |
| Calcium (mg/Day) | 184.53 (±111.10) | 189.27 (±121.23) | 175.87 (±155.34) |  |
| Oxalate (mg/Day) | 37.13 (±15.60) | 41.62 (±25.07) | 45.65 (±16.99) |  |
| Citrate (mg/Day) | 563.05 (±361.15) | 564.72 (±424.41) | 503.71 (±433.69) |  |
| Sodium (mmol/Day) | 177.49 (±69.23) | 187.74(±75.31) | 207.06 (±85.72) |  |
| Uric Acid (g/Day) | 0.63 (±0.25) | 0.70 (±0.26) | 0.75 (±0.33) |  |
| pH | 6.05 (±0.53) | 5.90 (±0.49) | 6.01 (±0.54) |  |
| CaOx Supersaturation | 5.73 (±2.43) | 5.94 (±3.40) | 5.80 (±2.88) |  |
| CaP Supersaturation | 0.74 (±0.71) | 0.72 (±0.81) | 0.58 (±0.92) |  |
| UA Supersaturation | 0.73 (±0.74) | 0.99 (±0.79) | 0.79 (±0.74) |  |

CaOx: Calcium Oxalate, CaP: Calcium Phosphate, UA: Uric Acid

**Supplementary Table 3.** Relationship of age, gender, and BMI with urinary analyte abnormalities on 24-hour urine collection

| Urine Parameter | Age Coefficient | Gender Coefficient | BMI Group Coefficient |
| --- | --- | --- | --- |
| Low Urine Volume | P=0.617 | **P<0.001** | P=0.565 |
| Hypercalciuria | **P<0.001** | **P<0.001** | **P<0.001** |
| Hyperoxaluria | P=0.656 | **P<0.001** | **P<0.001** |
| Hypocitraturia | P=0.228 | **P<0.001** | **P<0.001** |
| Elevated Urine Sodium | **P<0.001** | **P<0.001** | **P<0.001** |
| Hyperuricosuria | **P<0.001** | **P<0.001** | **P<0.001** |
| pH<5.5 | **P<0.001** | **P<0.001** | **P<0.001** |
| CaOx Supersaturation | P=0.930 | P=0.562 | P=0.704 |
| CaP Supersaturiaton | **P<0.001** | P=0.628 | P=0.735 |
| UA Supersaturiation | **P=0.032** | **P<0.001** | **P<0.001** |
| Coefficient significance (p-value) of a multivariable logistic regression analysis. CaOx: Calcium Oxalate, CaP: Calcium Phosphate, UA: Uric Acid | | | |
